# Prevalence and genotype distribution of group A rotavirus circulating in Shanxi Province, China during 2015–2019

**DOI:** 10.1186/s12879-021-05795-4

**Published:** 2021-01-21

**Authors:** Lifeng Zhao, Xiaohong Shi, Dequan Meng, Jiane Guo, Yiping Li, Lirong Liang, Xiaofang Guo, Ran Tao, Xiaohua Zhang, Ruihong Gao, Li Gao, Jitao Wang

**Affiliations:** 1 Department of Microbiology Test, Taiyuan Center for Disease Control and Prevention, NO. 89 Xinjian South Road, Taiyuan, 030012 Shanxi Province China; 2 Department of Disease Prevention and Public Health, Children’s Hospital of Shanxi, Taiyuan, 030001 Shanxi Province China; 3 Department of Tuberculosis Control and Prevention, Taiyuan Center for Disease Control and Prevention, Taiyuan, 030012 Shanxi Province China

**Keywords:** Group a rotavirus, Active surveillance, Acute gastroenteritis, Genotypes

## Abstract

**Background:**

Group A rotavirus (RVA), despite being a leading cause of gastroenteritis in infants and young children, is less studied in Shanxi Province, China. The current study was conducted to determine the prevalence and genetic characterization of RVA in hospitalized children younger than 10 years of age with the diagnosis of acute gastroenteritis in Shanxi Province, China.

**Methods:**

A hospital-based active surveillance of rotavirus gastroenteritis was conducted at Children’s Hospital of Shanxi from Jan 1, 2015, through Dec 31, 2019. Rotavirus was detected in stool samples by real-time quantitative reverse transcription PCR (qRT-PCR). G- and P-genotypes were determined by reverse transcription PCR (RT-PCR) and nucleotide sequencing.

**Results:**

A total of 961 children younger than 10 years of age was enrolled over the study period, of whom 183 (19.0%) were positive for RVA. The highest RVA-infection frequency (23.7%) was found among children aged 12–23 months, and the seasonal peak was in December. G9P[8] was most prevalent (76.0%), followed by G3P[8] (7.1%), G2P[4] (3.3%), G1P[8] (0.5%) and G9P[4] (0.5%).

**Conclusions:**

These results report for the first time that RVA was one of the main causes of severe infectious gastroenteritis in children, and a high proportion of G9P[8] strains circulating in most areas of Shanxi Province. While the protective efficacy of the rotavirus vaccines has been demonstrated against G9P[8] strains, our results highlight that the dominant strains have not been effectively controlled in China.

**Supplementary Information:**

The online version contains supplementary material available at 10.1186/s12879-021-05795-4.

## Background

Group A rotaviruses (RVAs) are the leading etiological agents of severe childhood gastroenteritis worldwide [1]. The virus is a nonenveloped and double-stranded RNA virus belongs to the *Reoviridae* family. Members of the genus *Rotavirus* encoding six structural proteins (VP1-VP4, VP6, VP7) and six nonstructural proteins (NSP1-NSP6). Two outer capsid proteins, VP7 (or G protein) and VP4 (or P protein) are considered to define the serotype; their coding genes are classified into multiple genotypes based on sequence diversity [2, 3]. Currently, more than 36 G and 51 P types have been detected and at least 60 combinations have been identified from humans and other animals [4–6]. Globally, the most common G-P genotype combinations causing 90% infections in children were G1P[8], G2P[4], G3P[8], G4P[8], G9P[8], and G12P[8] [2, 3]. Of these, their relative proportions may vary by year and region [7].

Viral gastroenteritis is a common infectious disease syndrome, causing a combination of nausea, vomiting, diarrhea, and abdominal pain [8]. Rotaviruses are the dominant pathogens and more than 95% of rotavirus gastroenteritis (RVGE) are caused by RVA [9]. Worldwide, according to WHO estimates in 2013, RVA was the leading cause of gastroenteritis death among children < 5 years old and caused ~ 215,000 deaths each year [10]. In China, a survey, based on 45 reports from 1994 through 2014, indicated that RVAs cause ~ 40% and ~ 30% of viral-diarrhea-related hospitalizations and outpatient visits, respectively [11]. Besides, gastroenteritis caused by RVA can be more severe than that caused by other diarrhea viruses (e.g., norovirus, astrovirus, sapovirus) [12]. In a hospital-based study, RVA-positive was associated with a higher proportion of abdominal pain, dehydration, fever, and a greater estimated rate of hospitalization than RVA-negative [13].

Given the prevalence and gravity, the rotavirus vaccines are expected to play a critical role in reducing the tremendous burden. In China, LuoTeWei, also known as LLR (Lanzhou Lamb Rotavirus vaccine, Lanzhou Institute of Biological Products, China), has long been the only used vaccine within the last two decades (2000–2018), which is an oral, live, attenuated rotavirus vaccine sourced from G10P[12] of sheep [14]. LuoTeWei is currently recommended in China as a single dose annually from 2 months to 3 years of age and a booster dose between 3 and 5 years [14]. At the end of 2018, RotaTeq (Merck Sharp & Dohme Products, USA) was available nationally and licensed for vaccinating children aged 6–32 weeks in China [15]. The effectiveness of any rotavirus vaccine is still unknown due to the comprehensive and continuous surveillance has not been carried out in China.

Up to now, the Rotavirus Surveillance System (RSS) was established only in regional cities such as Beijing, Jiangsu, Sichuan, and Guangdong. In Beijing and Guangdong, rotavirus continuous surveillance all began from 1998, which includes monitoring rotavirus test-positive rates and strain genotypes among inpatients younger than 5 years with diarrhea in selected sentinel hospitals [16]. In Sichuan and Jiangsu, except for rotavirus surveillance in children, there have been reports of rotavirus strains in animals [17–19]. Shanxi Province, located in the middle-area of China, is one of the regions with a serious burden of gastroenteritis infectiosa [20], but RVGE has long been ignored because it is considered to be a vaccine-preventable and self-limiting disease. Up to now, the incidence of rotavirus-infection has not been counted in the Chinese Disease Surveillance System (CDSS), so the genetic and epidemiological information of rotavirus is still unclear locally. Moreover, some rotavirus vaccines have been reported to be highly efficacious in reducing severe disease in many countries [21–23], but rotaviruses continue to evolve and novel strains continue to emerge, whether those are effective is still unknown.

In the current study, a hospital-based surveillance of rotavirus in pediatric inpatients has been first established in 2015 in Shanxi Province, China. The specific aims were to estimate the disease burden, genotype distribution, and thus inform targeted control measures and vaccine development.

## Methods

### Study area

Shanxi Province is located in the middle-area of China (latitude 34°34′–40°44′N and longitude 110°14′–114°33′E), consisting of 11 cities (80 counties) and a population of about 37 million.

### Specimens

Fecal specimens were collected from children < 10 years old who were hospitalized for diarrhea in Children’s Hospital of Shanxi (CHS) between Jan 1, 2015, and Dec 31, 2019. Oral informed consent was obtained from their patients or guardians. All enrolled children have obvious symptoms of gastroenteritis (defined as ≥3 loose stools over a 24 h period), and only those who have not received any medications and medical tests before hospitalization were eligible for enrolment. Patients exceed the age and diagnosed with chronic diarrhea by clinicians were excluded from this study. Specimens were collected in a sterile sampling cup, keeping low temperature (4 °C), and were sent to the virus microbiology laboratory of Taiyuan Center for Disease Control and Prevention (Taiyuan CDC) for rotavirus nucleic acid testing within 24 h. Upon receipt, each sample was allocated a unique laboratory code and entered into a gastroenteritis information database (Excel) in Taiyuan CDC.

### RNA extraction

Fecal suspensions (10%, w/v) were prepared of 0.01 M phosphate-buffered saline (PBS) (pH = 7.2), vortexed, and centrifuged (8000 g at 4 °C for 8 min). Total RNA was extracted from 60 μL of the fecal suspension using a MagMax-96 Viral RNA Isolation Kit (Thermo Fisher Scientific, Foster City, CA), according to the manufacturer’s instructions. RNA-positive control and negative control (PBS) was included in the extraction procedure in each batch, and the quality of extracted RNA was checked through a NanoDrop 1000 Spectrophotometer (NanoDrop Technologies, Houston, TX, USA). Rotavirus nucleic acid testing and genotyping were kept physically separated. All steps of sample preparation and RNA extraction were done in a biosafety cabinet.

### Detection of rotavirus

The real-time quantitative reverse transcription PCR (qRT-PCR) was applied by using a Rotavirus (Group A, B, and C) Multiple Real-time PCR Kit (TaqMan probe) (S-SBIO, Taizhou, China). PCR cycling parameters were set up according to the instruction: 50 °C for 30 min, 95 °C for 5 min, followed by 45 cycles of 94 °C for 10 s, 55 °C for 40 s in a CFX96 Real-time Thermal Cycler (Bio-Rad, Hercules, CA). A positive result was defined as a threshold cycle (Ct) value < 35, and positive internal control was defined as a Ct value < 30. The qRT-PCR negative samples were not under the scope of our study.

### Gene amplification and nucleotide sequencing

For *VP7* amplification, the reverse transcription PCR was carried out using a PrimeScript One Step RT-PCR Kit. (TaKaRa, Dalian, China). A 1062-bp fragment was amplified with the consensus forward primer *Beg9* (*GGCTTTAAAAGAGAGAATTTCCGTCTGG*) and the reverse primer *End9* (*GGTCACATCATACAATTCTAATCTAAG*). The amplification conditions included: 50 °C for 30 min, 94 °C for 2 min followed by 32 cycles of 94 °C for 30 s, 55 °C for 30 s, 72 °C for 90 s, and a final extension of 72 °C for 5 min. For the *VP4* amplification, the semi-nested reverse transcription PCR was carried out using a PrimeScript One Step RT-PCR Kit. The first amplification was carried out using primers *VP4F2* (*TTTATAGACAGCTTCTCACTAATTC*) and *VP4R3* (*TATGTGCAGTTACTTGTTCACC*) under the condition of 50 °C for 30 min, 94 °C for 2 min, followed by 10 cycles of 94 °C for 30 s, 55 °C for 30 s, 72 °C for 90 s. Then 1 μl of the first PCR products was used for amplification with the nested primers *VP4F4* (*TTTATAGACAGCTTCTCACTAATTC)* and *VP4R3* (*TATGTGCAGTTACTTGTTCACC*). The amplification conditions included 94 °C for 5 min, followed by 30 cycles of 94 °C for 30 s, 55 °C for 30 s, 72 °C for 1 min, and a final extension of 72 °C for 5 min. A portion (5 ul) of the reaction mixture with loading buffer, followed electrophoresis in 1.5% agarose gel (Takara, Dalian, China), was visualized by Goldview (Transgen, Beijing, China). The amplicons were purified and sequenced by Sangon Biotech (Shanghai, China). All the PCR runs included the positive control and non-target control (reagent blank) to avoid false-positive results. All the primers were synthesized by Sangon Biotech (Shanghai, China).

### Determination of RVA genotypes and phylogenetic analysis

The resulting sequences were prepared and aligned by BioEdit (version 7.2.5) with the Clustal-W program. Based on the sequence data, the genotype assignment was accomplished using BLAST (http://blast.ncbi.nlm.nih.gov/Blast.cgi) and RotaC v2.0 (http://rotac.regatools.be). Phylogenetic trees were constructed using the Neighbor-joining method with MEGA (version 5.0) and bootstrap analysis was performed with 1000 replications. The reference sequences used in plotting the phylogeny trees from the GenBank database are shown in Table 1.

**Table 1:** Reference sequences used in plotting the phylogenetic tree of this study

Accession number	Strains	Date	Location	Genotypes	Accession number	Strains	Date	Location	Genotypes
KU243671	WZ202	2016	Zhejiang (China)	G1	MG816527	SC6	2013	Sichuan (China)	G9
KX009876	Kerala-RV01	2013	India	G1	KX778608	km15119	2016	Yunnan (China)	G9
GU565057	RotaTeq-WI79–9	1992	USA	G1	KT919508	VU12–13-101	2013	USA	G9
HM130956	KR/Seoul-710	2009	Korea	G2	LC477377	Tokyo18–43	2018	Japan	G9
LC477357	Tokyo17–10	2017	Japan	G2	AB180971	A2	19xx^a^	USA	G9
GU565068	RotaTeq-SC2–9	1992	USA	G2	KX363355	14,150	2012	Vietnam	G9
KF371856	E2432	2010	Hubei (China)	G3	MF139499	CU192	2016	Thailand	G9
LC477355	Tokyo17–08	2017	Japan	G3	KU243609	WZ189	2013	Zhejiang (China)	P[4]
KY661928	1CR7	2015	Thailand	G3	MG729831	Hu/13–146	2013	Shanghai (China)	P[4]
GU565079	RotaTeq-WI78–8	1992	USA	G3	KF372017	Z1602	2012	Hubei (China)	P[8]
EU348715	P50	20xx^a^	Slovenia	G3	KX778584	Km15119	2016	Yunnan (China)	P[8]
KX911619	CU140	2016	Thailand	G3	MG816520	SC1	2014	Sichuan (China)	P[8]
L35055	A131	1988	Venezuela	G3	MF580855	Hu/JS2012	2012	Jiangsu (China)	P[8]
KF673479	BJ-Q322	2011	Beijing (China)	G9	LC477409	Tokyo18–50	2018	Japan	P[8]
KF673482	BJ-Q794	2012	Beijing (China)	G9	GU565044	RotaTeq-WI79–4	1992	USA	P[8]
MF580843	Hu/JS2013	2013	Jiangsu (China)	G9					

Note: ^a^ There are missing data

### Nucleotide sequence accession numbers

The nucleotide sequences used in this study were submitted in GenBank (https://www.ncbi.nlm.nih.gov/genbank/) under accession numbers MT710743 - MT710824 (VP7) and MT710825 - MT710927 (VP4), which are shown in Table S2.

### Statistical analysis

Data analyses were performed by IBM SPSS Statistics software (Version 19.0). Univariate logistic regression was performed to ascertain the equality of means of RVA positive or negative for variables: sexes, symptoms, and sample types (Table 2). Statistical significance was defined as *p* < 0.05.

**Table 2:** Sexual distinction, symptoms, and sample types of this study

Demographics	Total	RVA-positive (n)	RVA-negative (n)	RVA-positive (%)	*P*-value
Gender					
Male	609	114	495	18.72	
Female	352	69	283	19.60	0.46
Symptom (Diarrhea with)					
vomiting	213	51	162	23.9	0.13
fever	403	92	311	22.8	0.02
abdominal pain	38	3	35	7.9	0.13
dehydration	17	3	14	17.6	0.61
Sample type					
Watery stool	663	145	518	21.9	0.84
Loose stool	151	22	129	14.6	0.79
Purulent bloody stool	74	8	66	10.8	0.59
Mucus stool	67	7	60	10.4	0.54

## Results

### Patients with gastroenteritis

**Fig. 1:**
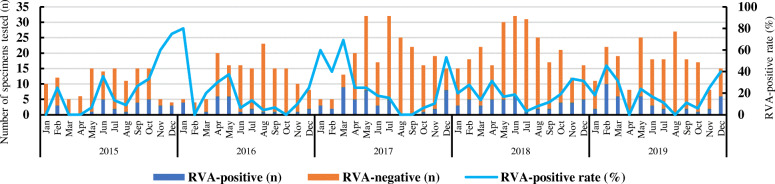
Monthly distribution and rotavirus positivity among hospitalized children under ten years old with gastroenteritis in Shanxi Province, China, 2015–2019

During the period 2015–2019, a total of 961 hospitalized children younger than 10 years of age, 609 boys and 352 girls, confirmed providing fecal specimens for rotavirus testing. RVA was detected in 183 stool samples via qRT-PCR with an overall positivity rate of 19.0% (183/961). Rotavirus B and C were not detected. The maximum number of samples was tested in May 2017, and the largest number of RVA-positive cases occurred in February 2019 (Fig. 1). No statistically significant difference was found between sex and RVA-infection (*p* > 0.05) (Table 2). Types of fecal specimen collected in this study included watery stool, loose stool, pus and blood stool, and mucus stool, no statistically significant difference existed between four types positive for RVA (Table 2). Apart from the symptom of diarrhea, some patients were accompanied by other related symptoms such as vomiting, fever, abdominal pain, and dehydration. There was a statistically significant difference between RVA-infection and fever (> 38.5 °C) (*p* < 0.05). Whereas, no significant difference (*p* > 0.05) was found between RVA-infection and the other three symptoms (vomiting, abdominal pain, and dehydration) (Table 2). Twenty RVA-positive patients coinfected with other diarrhea associated viruses: 16 with norovirus, 2 with sapovirus, and 1 with astrovirus. One was coinfected with more than two viruses (norovirus and astrovirus). Six were coinfected with other pathogenic bacteria: 2 with Salmonella, 3 with EAEC, and 1 with ETEC (Table S1).

### Age, geographical and seasonal features

**Fig. 2:**
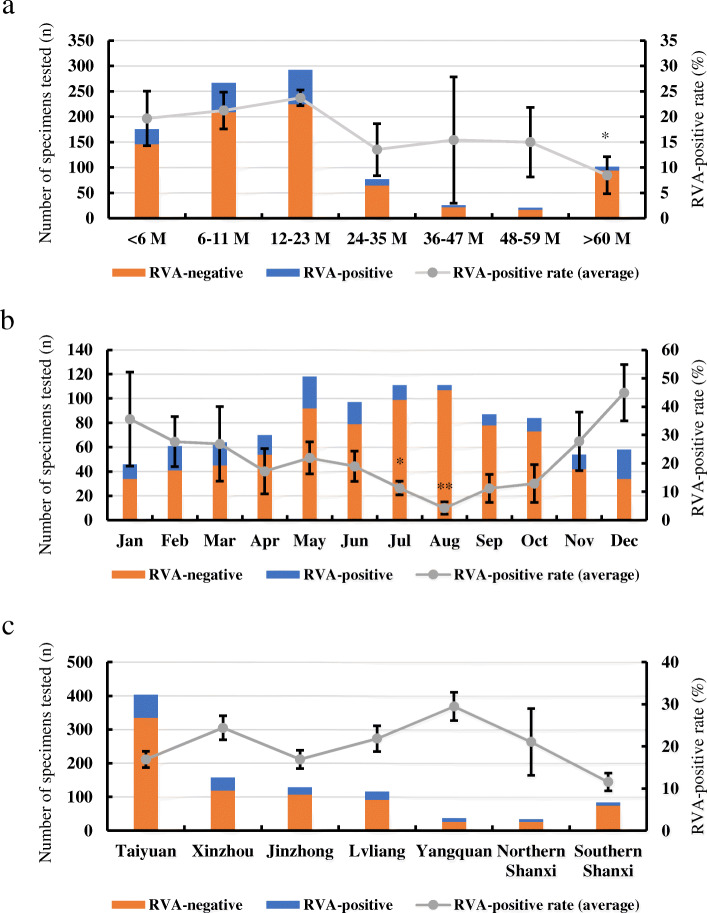
Prevalence of RVA infections in Shanxi Province, China, 2015–2019. **a** Age distribution; **b** Monthly distribution; **c** Geographical distribution. *P* values: * *p* < 0.05, ** *p* < 0.01. Error bars: standard deviation

Overall, most cases (292) were found in the age group of 12–23 months which was also the group with the highest average RVA-positive rate (23.7%) (Fig. 2a). On the contrary, the lowest rate (8.5%) was observed in the group > 60 months with a statistically significant difference (*p* < 0.05, Fig. 2a). More than one-fifth of gastroenteritis cases were found in July–August, but the RVA-positive rate remained low and with a statistically significant difference (*p* < 0.05, Fig. 2b). 44.9% of the RVA-positive cases were found in December, although gastroenteritis cases were rare in the cold season (November–January) (Figs. 1 and 2b). All the patients came from the cities or regions of Shanxi Province, the highest prevalence (29.5%) was observed in Yangquan, followed by 22.4% in Xinzhou, 21.8% in Lvliang, 21.1% in Northern Shanxi, and 16.9% in Taiyuan (Fig. 2c). The patients from Southern Shanxi had the lowest average positive rate (11.6%, Fig. 2c).

### *VP7* and *VP4* genotyping

**Fig. 3:**
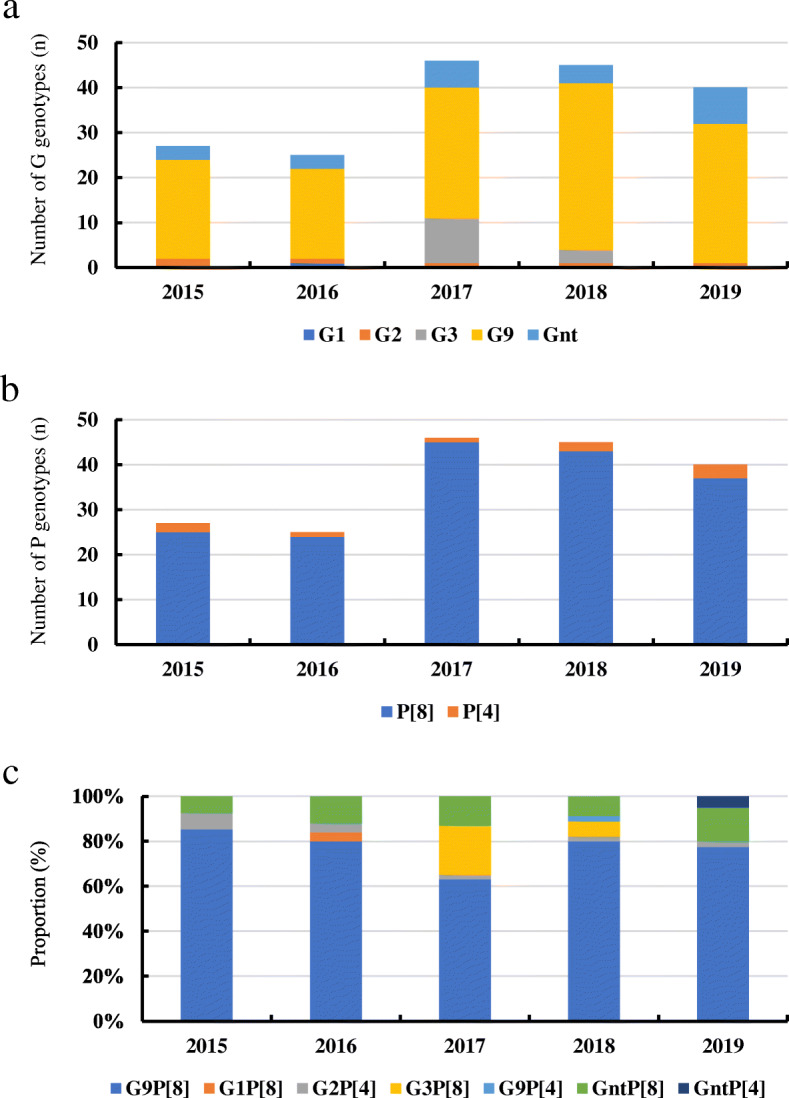
Prevalence of different RVA genotypes by surveillance year in Shanxi province, China, 2015–2019. **a** G genotypes; **b** P genotypes; **c** Proportions of different genotypes

One hundred fifty-nine samples were genotyped for *VP7*, and 183 samples were successfully genotyped for *VP4*. G9 was the prevalent G-genotype, representing 76.0% (139/183) of all positive samples, followed by G3 (7.1%, 13/183), G2 (3.3%, 6/183), and G1 (0.5%, 1/183) (Fig. 3a). P[8] (95.1%, 174/183) was the prevalent P-genotype, followed by P[4] (4.9%, 9/183) (Fig. 3b). One hundred fifty-nine strains were confirmed both G- and P-genotype. G9P[8] was the most commonly detected genotype (76.0%, 139/183), followed by G3P[8] (7.1%, 13/183), G2P[4] (3.3%, 6/183), G1P[8] (0.5%, 1/183), and G9P[4] (0.5%, 1/183) (Fig. 3c).

### Geographical distribution

**Fig. 4:**
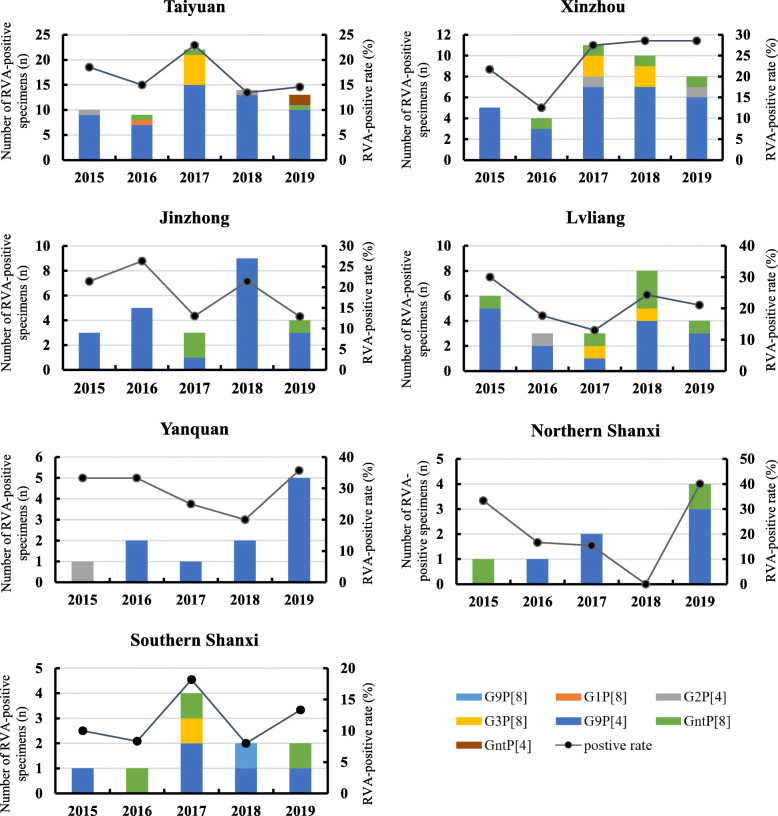
Geographical distribution of different RVA genotypes in cities or regions of Shanxi Province, China, 2015–2019

Due to the long distance between the patients and the designated hospital, cases from two northern cities (Datong and Shuozhou) and four southern cities (Linfen, Yuncheng, Changzhi, and Jincheng) were less compared with the central cities of Shanxi Province, therefore, cases from these cities were divided into two independent parts (Northern Shanxi and Southern Shanxi) for next analyzing. G9P[8] was the dominant genotype and occupied more than 80% of RVA positive cases in most cities or regions of Shanxi Province (Fig. 4). The only G1P[8] and G9P[4] were detected in Taiyuan (2016) and Southern Shanxi (2018), respectively (Fig. 4). G2P[4] was detected in Taiyuan, Xinzhou, Lvliang, and Yanquan; G3P[8] was detected in Taiyuan, Xinzhou, Lvliang, and Southern Shanxi. Strains that cannot be typed (GntP[8] and GntP[4]) were found in most areas in Shanxi province, except Yangquan (Fig. 4).

### Phylogenetic analyses of VP7 genes

**Fig. 5:**
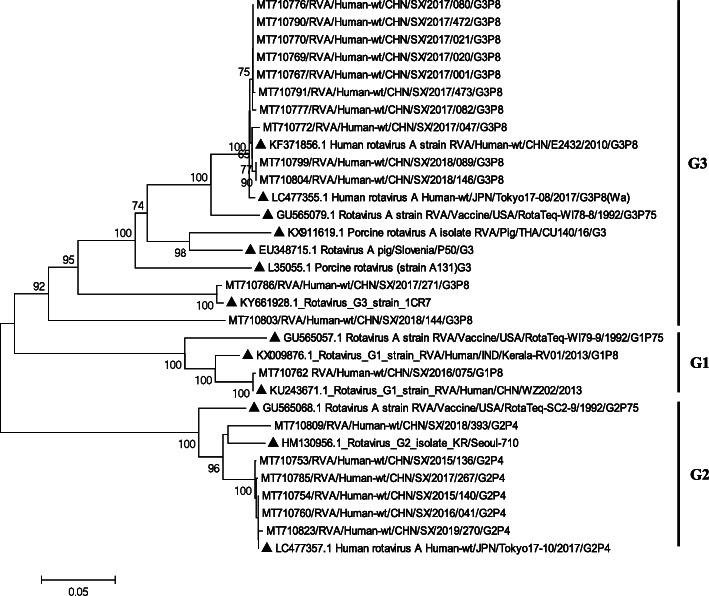
Neighbor-joining phylogenetic tree constructed from the partial VP7 genes (798 bp) of G1, G2, and G3 strains, and representative RVA strains with the kimura-2-parameter model in MEGA program 5.0. Bootstrap values estimated with 1000 replicate data sets were indicated at each node. The scale bar indicated the number of nucleotide substitutions per site. Bootstrap values lower than 60% are not shown. The reference strains are represented by the triangle. The RVA strains sequenced in this study are represented by Accession number/Country/Province/Year/Serial number/Genotype

The G1-G2-G3 VP7 tree was constructed based on 18 Shanxi strains sequenced in this study and 13 representative members (Fig. 5, Table 1). The G1 lineage contained the only Shanxi strain (SX/2016/073) and two representative strains (WZ202 and Kerala-RV01) detected in China and Indonesia with 95.9 and 99.8% nucleotide similarity, respectively. SX/2016/073 shared 90.8% nucleotide similarity and 92.8% amino acid similarity with the RotaTeq G1 strain RotaTeq-WI79–9 (Fig. 5, G1). Five Shanxi G2 strains (SX/2015/136, SX/2017/267, SX/2019/270, SX/2014/140, and SX/2016/011) were closely related to a Japanese strain (Tokyo 17–10) detected in 2017, with 99.8–99.9% nucleotide similarities. The other Shanxi G2 strain (SX/2018/393) was related to a South Korea strain (Seoul-710) with a nucleotide similarity of 94.9%. All the Shanxi G2 strains shared 92.7–92.9% nucleotide similarity and 95.1–95.5% amino acid similarity with the RotaTeq G2 strain RotaTeq-SC2–9 (Fig. 5, G2). Ten Shanxi G3 strains (SX/2017/001, SX/2017/021, SX/2017/080, SX/2017/172, SX/2017/020, SX/2017/473, SX/2017/082, SX/2017/047, SX/2018/089, and SX/2018/146) were closely related to the representative strains (E2432 and Tokyo 17–08) with high nucleotide similarities (99.3–99.6%). The other Shanxi G3 strain (SX/2017/271) was related to 1CR7 detected in South Korea (2017), with 99.3% nucleotide similarity. All the Shanxi G3 strains shared 81.8–94.1% nucleotide similarity and 92.5–97.7% amino acid similarity with the RotaTeq vaccine G3 strain RotaTeq-SC2–9 (Fig. 5, G3).

**Fig. 6:**
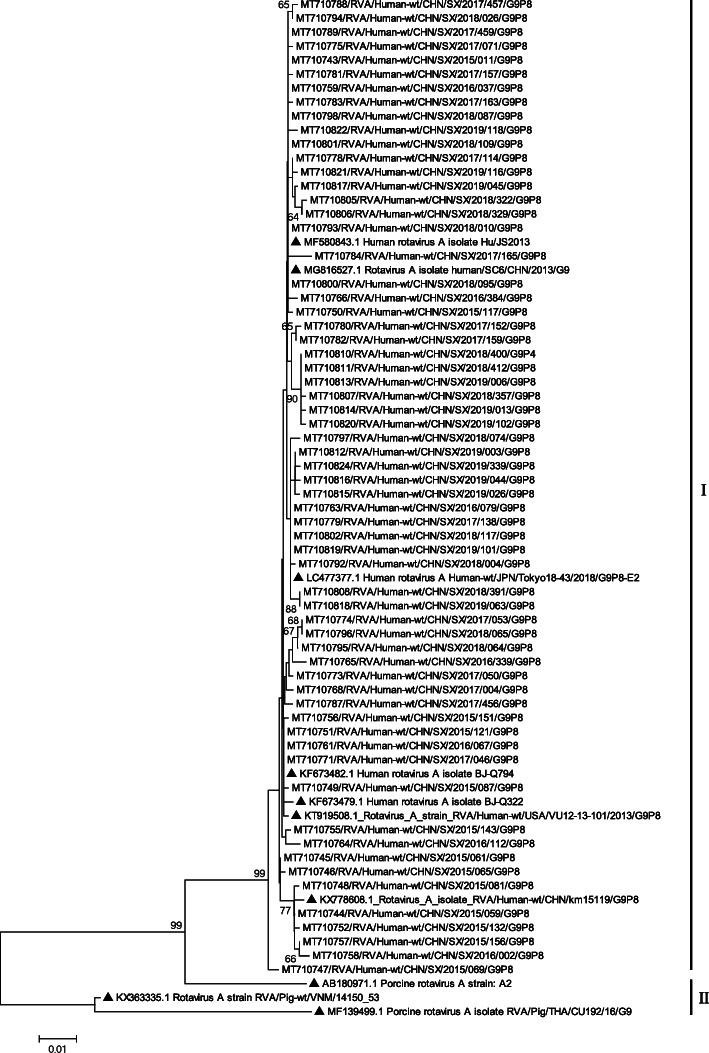
Neighbor-joining phylogenetic tree constructed from the partial VP7 genes (798 bp) of G9 strains and representative RVA strains with the kimura-2-parameter model in MEGA program 5.0. Bootstrap values estimated with 1000 replicate data sets were indicated at each node. The scale bar indicated the number of nucleotide substitutions per site. Bootstrap values lower than 60% are not shown. The reference strains are represented by the triangle. The RVA strains sequenced in this study are represented by Accession number/Country/Province/Year/Serial number/Genotype

Sixty-three Shanxi G9 strains and 10 representative members were selected for G9 phylogenetic analyses (Fig. 6, Table 1). Except for SX/2015/069, 62 Shanxi G9 strains fell into 2 minor lineages (I and II). Fifty-five Shanxi G9 strains (belong to lineage 1) were closely related to each other with 99.2–99.9% nucleotide similarity, clustering with other strains isolated in Japan (Tokyo 18–43), USA (VU12–13-101), Thailand (T152), and China (SC6, Hu/JS2013, BJ-Q794) during 2011–2018 (Fig. 6, lineage I). Seven Shanxi G9 strains (SX/2015/061, SX/2015/065, SX/2015/081, SX/2015/059, SX/2015/132, SX/2015/156, and SX/2016/002) belong to lineage II were closely related to each other with 99.4–99.9% nucleotide similarity, clustering with a Chinese strain (km15119) isolated in Yunnan Province in 2016 (Fig. 6, lineage II). The SX/2015/069 was separate from other Shanxi G9 strains, but still has 99.2% nucleotide similarity with the reference strain HU/JS2013 (Fig. 6).

### Phylogenetic analyses of VP4 genes

**Fig. 7:**
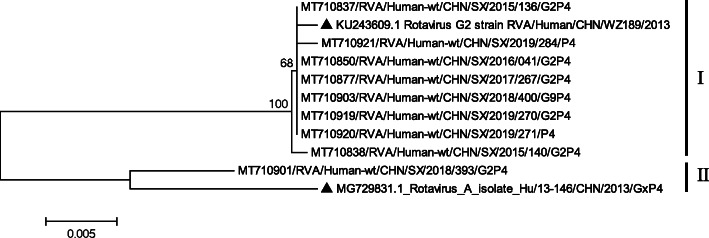
Neighbor-joining phylogenetic tree constructed from the partial VP4 genes (696 bp) of P[4] strains and representative RVA strains with the kimura-2-parameter model in MEGA program 5.0. Bootstrap values estimated with 1000 replicate data sets were indicated at each node. The scale bar indicated the number of nucleotide substitutions per site. Bootstrap values lower than 60% are not shown. The reference strains are represented by the triangle. The RVA strains sequenced in this study are represented by Accession number/Country/Province/Year/Serial number/Genotype

The P[4]-VP4 tree was constructed based on 9 Shanxi strains and 2 representative members (Fig. 7, Table 1). Eight Shanxi strains (SX/2015/136, SX/2019/271, SX/2019/284, SX/2018/400, SX/2019/270, SX/2016/041, SX/2017/267, and SX/2015/140) were closely related to WZ189 detected in Zhejiang Province (China) in 2017, with high nucleotide similarities (99.7–99.9%). The other Shanxi strain SX/2018/393 was closely related to the Hu/13–146 detected in Shanghai (China), with 98.0% nucleotide similarity (Fig. 7).

**Fig. 8:**
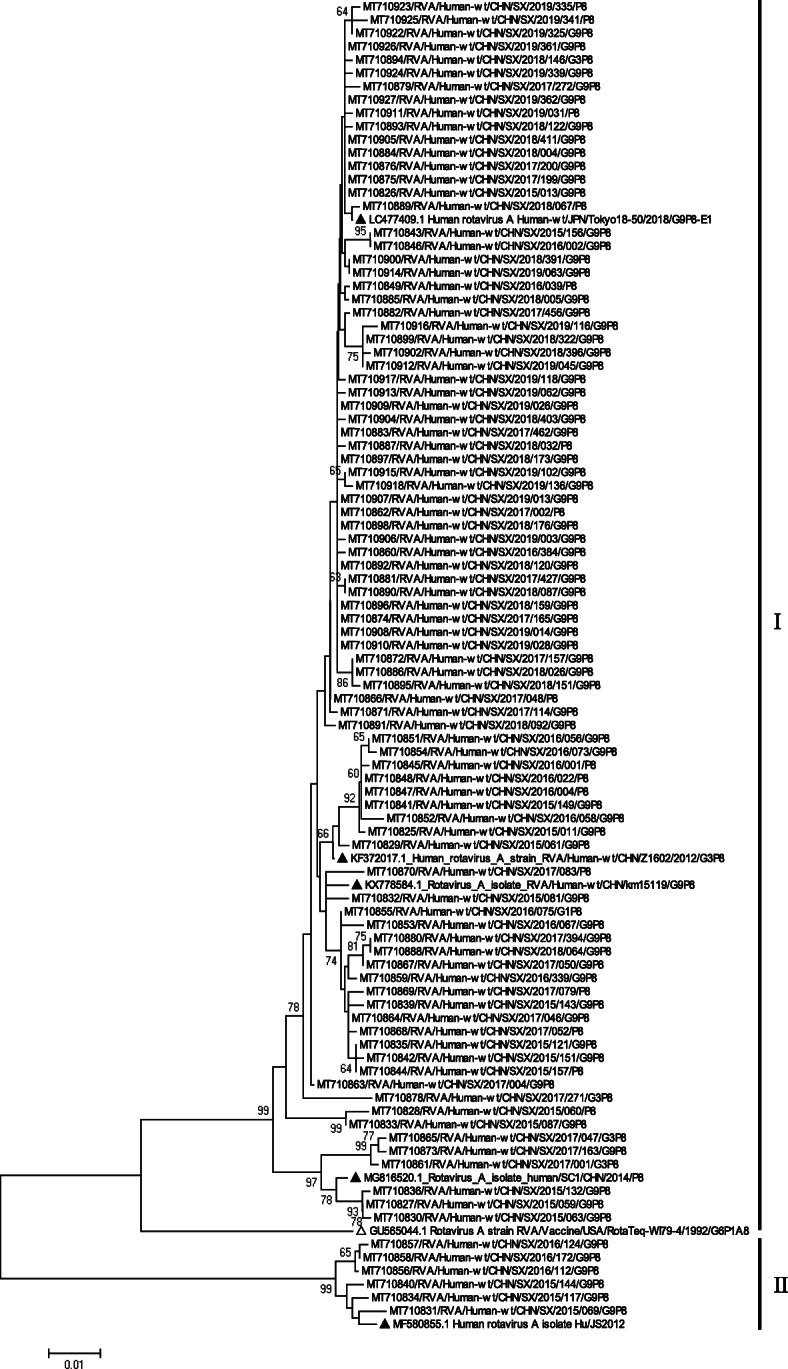
Neighbor-joining phylogenetic tree constructed from the partial VP4 genes (696 bp) of P[8] strains and representative RVA strains with the kimura-2-parameter model in MEGA program 5.0. Bootstrap values estimated with 1000 replicate data sets were indicated at each node. The scale bar indicated the number of nucleotide substitutions per site. Bootstrap values lower than 60% are not shown. The reference strains are represented by the triangle. The RVA strains sequenced in this study are represented by Accession number/Country/Province/Year/Serial number/Genotype

The P[8]-VP4 tree was constructed based on 93 Shanxi strains and 6 representative members (Fig. 8, Table 1). The Shanxi G9 strains fell into 2 minor lineages (I and II, Fig. 8). Eighty-seven Shanxi P[8] strains (belong to lineage 1) were closely related to each other with 96.9–99.9% nucleotide similarity, clustering with a Japanese strain (Tokyo 18–50) and 3 China’s strains (Z1602, km15119, SC1) isolated in previous studies during 2011–2018. The Shanxi P[8] strains belong to lineage I shared 91.9–93.0% nucleotide similarity and 93.1–94.0% amino acid similarity with the RotaTeq vaccine P1a[8] strain RotaTeq-WI79–4 (Fig. 8, lineage I). The other 6 Shanxi P[8] strains (SX/2016/124, SX/2016/172, SX/2016/112, SX/2015/144, SX/2015/117, and SX/2015/069) belong to lineage 2 were closely related to a Chinese strain Hu/JS-2012 detected in Jiangsu Province in 2012, with 98.9–99.3% nucleotide similarities (Fig. 8, lineage II). The Shanxi P[8] strains belong to lineage II shared 87.6–87.7% nucleotide similarity and 88.4–88.8% amino acid similarity with the RotaTeq vaccine P1a[8] strain RotaTeq-WI79–4 (Fig. 8).

## Discussion

Infecting nearly every child, human group A rotaviruses have been proven to be a major cause of pediatric diarrhoeal disease morbidity and mortality all through the world [24].

In the current study, the RVA-positive rate found in children < 10 years old in Shanxi Province was 19.0%, which is comparable with the results of 20.8 and 17.5% in Beijing (2011–2016, 15] and Chengdu (Sichuan Province, China, 2009–2014) [25], respectively. However, a sentinel-based surveillance of diarrhea study by Zeng in Chongqing found a prevalence of 30.5% in the period of 2011–2015 [13], which is much higher than the current study. There might be several reasons, such as different patient categories (outpatient or inpatients), different definitions of gastroenteritis, selection of suspected cases, types of the sample tested (serum, plasma, or feces), the standard for valid sample, and types of diagnostic reagent (ELISA or RT-PCR). Specifically, among children under 5 years old, the positive rate in Shanxi Province is 20.4% (175/859). Compared with the reports from other WHO regions where rotavirus vaccines have been introduced after 2008, the average proportion of acute gastroenteritis cases positive for rotavirus in our study was lower than African Region, Region of the Americas, Eastern Mediterranean Region, and higher than European Region [26].

In this study, up to one-half of inpatients have febrile symptoms (> 38.5 °C), and more than one quarter have a symptom of vomiting (Table 2). However, it is not possible to diagnose RVA infection by clinical presentation because the clinical features of RVGE do not differ from those caused by other pathogens. Severe, lethal RVA infections did not appear among all the RVA-positive cases, which may be different from Liu’s research [11]. During the whole process, based on the data of routine blood tests, we found that the proportion of viral infections was significantly higher than bacterial infections (data not shown). However, the abuse of antibiotics has not been effectively controlled in China, and most gastroenteritis patients have used antibiotics before seeing a doctor. Therefore, it is impossible to prove that virus is the main cause of child gastroenteritis compared with bacteria in Shanxi Province.

Available data suggest that RVA is susceptible to people of any age but spreading more easily among infants and young children [27]. Studies in Shanghai had discovered that the RVA prevalence was higher in children aged 3–5 years [28]. In our study, RVA-infection mainly occurs in children under 2 years of age with a peak positive rate in children of 12–23 months, followed by 6–11 months, and 0–6 months (Fig. 2a), which was similar to a Beijing’s study [15]. Considering the highest positive rate was in the 0–23-month age group, early introduction of rotavirus vaccination to children is desirable.

Globally, there are obvious seasonal and geographical variations in RVA prevalence. In this study, RVA infections occurred throughout the year, but with the highest detection rate in the cold season (Fig. 2b). This finding is identical to a monitoring analysis by Yu et al. on the seasonality of RVA infection [29]. The same patterns have also been reported in other Asian countries like Pakistan [30] and India [27]. However, according to the WHO, RVA prevalence was reported throughout the year in the subtropical zone and peaks often occur in dry seasons, unlike in the northern hemisphere countries where peaks occur in winter [31]. In Benin, West Africa, a higher rate of RVA-infection was recorded during the rainy season and the long dry season [31].

Molecular epidemiology studies have revealed that the distribution of RVA strains varies over both space and time. In China, G1/G3/G9-P[8] and G2P[4] were most commonly reported in the epidemiological studies from 1994 to 2013 [15]. According to the existing research results, the formation of dominant genotypes is a long-term evolutionary process. In 2011, the genotype G3P[8] with G9P[8] was first found to be both the dominant genotypes in Beijing [15]. In 2015, Yu reported the predominant strain of RVA has rapidly evolved from G3P[8] and G1P[8] to G9P[8], with the proportion of G9P[8] having increased remarkably from 3.4% (2009) to 60.9% (2015) [29]. However, our results are significantly different from those of other countries in the last 10 years. In India, a study about RVA diversity showed that G1P[8] was still the predominant genotype among infants and children during 2012–2016 [27]. In 2014, G Ianiro reported that G1P[8] and G9P[8] strains were detected frequently from 2012 to 2014 in Italy, whereas G12P[8] caused a single large nosocomial outbreak [32]. These findings suggest that the G1, G2, and G3 strains, which represented the most prevalent genotypes worldwide in the past, are no longer the dominant strains in China. Besides, 24 strains (13.1%, 24/183) could not be genotyped (VP7) using the primers (*Beg9* and *End9*). There may be genetic mutations related to these strains, and the whole genome sequencing is needed for sequence analysis.

The behavioral characteristics and spatial transition of the floating population are important factors in RVA transmission. Taiyuan, the capital city of Shanxi Province, has the largest inhabitants and mobile residents, also contribute to the largest number of RVA-positive cases and a variety of genotypes. Cities adjacent to Taiyuan, such as Xinzhou, Jinzhong, and Lvliang, showed similar genotype distribution, but the genotype diversity was significantly lower than that in Taiyuan. In contrast, some cities or regions, with a small population and low mobility, have fewer kinds of genotypes. For example, only two genotypes were detected in Yangquan, and G9P[8] was the only genotype that was detected after 2015 (Fig. 4, Yangquan). Besides, based on relatively few case statistics, our study found that the RVA genetic diversity in the north part of Shanxi was significantly less than that in the south, which may be related to the population density and economic development level.

Phylogenetic trees were used to represent the evolutionary history and diversification of species. Our results indicate that most RVA strains circulating in Shanxi Province have high genetic homology with those isolated from other provinces in China. Worldwide, although the dominant genotypes may be different in the same period, the Shanxi strains also maintain high genetic similarity with those isolated from China’s neighboring countries, such as Japan, Thailand, and Pakistan. These results indicate that the genetic evolution of RVA strains occurs synchronously in certain areas, especially those areas with frequent population shifts.

Up to now, more than 20 genotypes have been identified from animals [4]. Our study found the Shanxi G3 strains were in the same evolutionary branch with two pig strains (CU140 and P50) isolated from Thailand and Slovenia, and the Shanxi G9 strains were in the same evolutionary branch with a pig strain (A2) isolated from the USA (Figs. 5, 6). These results imply that the Shanxi strains may have a common ancestor with the porcine rotavirus. However, it must be admitted that there are fewer rotavirus strains found in animals, so these conclusions may be overturned by in-depth research with the discovery of more rotavirus strains in animals.

Phylogenetic trees in this study were also used to evaluate the match between vaccine strains and Shanxi strains. Currently, only two rotavirus vaccines, LuoTeWei (G10P[12]) and RotaTeq (G1-G4, and P1a[8]), were licensed in China. Both vaccines have different genome compositions and antigenic characteristics, also provide broad heterotypic protection against other less common genotypes [33]. So far, all of them have not yet been included in the China National Immunization Program, and there were less available inoculation rate data reported officially. In 2018, Liu et al. investigated the rotavirus vaccination status of infants and young children born from 2008 to 2012 in six China’s provinces (Jiangsu, Chongqing, Guangdong, Jiangxi, Heilongjiang, and Gansu), and found the proportions of the first and the second dose of rotavirus vaccination (LuoTeWei) were 32.8 and 9.7%; Among the children vaccinated with the first dose, the vaccination age mainly concentrated in 2 to 21 months, of which the peak was 5 to 13 months [34]. Another study was conducted in Guangdong Province in 2013, He et al. reported an overall LuoTeWei vaccine coverage of 25.3% among children aged 2–59 months [35].

Our results confirmed that the vaccine genotypes G4, G10, and P[12] of the LuoTeWei and RotaTeq were not found in Shanxi Province. Beyond that, the G1, G2, G3, and P[8] strains in this study were related to the RotaTeq vaccine strains: RotaTeq-WI79–9(G1), RotaTeq-SC2–9(G2), RotaTeq-WI78–8(G3), and RotaTeq-WI79–4 (P1a[8]), respectively. However, there were about 7–12% differences in nucleotide and 3–11% in amino acid between the RotaTeq vaccine strain and Shanxi strains detected in this study. Our results also show that G9P[8] strains, which accounted for the highest proportion each year, had not been effectively controlled by the existing vaccines. The reason for this phenomenon may be the circulating strains cannot be effectively immunized by existing vaccines, or low inoculation rates. However, based on Merck’s data, RotaTeq is effective in the prevention of G9 rotavirus in a European study [33]. Due to the RotaTeq has been used locally for a short time, our results may not be used as a basis for evaluating the effectiveness.

This study has some limitations. First, only inpatients who have severe symptoms and need to be hospitalized for gastroenteritis were included in this study, the outpatients and those visiting the emergency department were not included. The positivity and genotypes of RVA apparently can be applied only to those with relatively high severity. Second, the effectiveness of a vaccine cannot be evaluated only by analyzing whether the genotype of the rotavirus vaccine matches the locally circulating strains. Furthermore, long-term ongoing surveillance is critical for evaluating interventions for RVA infections prevention and the use of phylogenetic analysis will be important to provide further insight into the impact of rotavirus vaccines on strain diversity.

## Conclusions

This study highlights that the rotavirus G9P[8] strains have been predominantly circulating locally for a long time in Shanxi Province, China. Although it provides only the hospitalized children epidemiological data, it will provide comprehensive knowledge to the public health authorities to calculate the RVA related disease burden and assess the effectiveness of the rotavirus vaccine on these emerged RVA strains in China.

## Supplementary Information

**Additional file 1: Table S1.** Coinfections in inpatients with RVGE. **Table S2.** GenBank accession numbers assigned for all rotavirus genotypes based on gene segment VP4 and VP7 sequenced in this study.

## Data Availability

The datasets used and analyzed during the current study are available from the first author and the corresponding author on reasonable request.

## Abbreviations

RVA: Group A rotavirus
RVGE: Rotavirus gastroenteritis
CHS: Children’s Hospital of Shanxi
CDC: Center for Disease Control and Prevention
qRT-PCR: Real-time quantitative reverse transcription polymerase chain reaction
