# Supplementary material for: Prevalence and genotype distribution of group A rotavirus circulating in Shanxi Province, China during 2015–2019
Source: BMC Infect Dis. 2021 Jan 21;21:94. doi: 10.1186/s12879-021-05795-4 (PMC7818068; doi:10.1186/s12879-021-05795-4)
Supplement: Supplementary file 1 — Additional file 1: Table S1. Coinfections in inpatients with RVGE. Table S2. GenBank accession numbers assigned for all rotavirus genotypes based on gene segment VP4 and VP7 sequenced in this study. [file 12879_2021_5795_MOESM1_ESM.docx]

Table S1. Coinfections in inpatients with RVGE

| \| Pathogens \| \| --- \| | No. (%) | Age group (Months) | | | | | | |
| --- | --- | --- | --- | --- | --- | --- | --- | --- | --- |
|  |  | <6 M | 6-11 M | 12-23 M | 24-35 M | 36-47 M | 48-59 M | >60M |
| Total | 183 |  |  |  |  |  |  |  |
| \| RVA + other enteric virus \| \| --- \| | 20 (10.9) | 6 | 7 | 7 | 0 | 0 | 0 | 0 |
| + Norovirus | 16 (8.7) | 5 | 6 | 5 | - | - | - | - |
| + Astrovirus | 1 (0.5) | 1 | - | - | - | - | - | - |
| + Sapovirus | 2 (1.1) | - | 1 | 1 | - | - | - | - |
| + Norovirus + Astrovirus | 1 (0.5) | - | - | 1 | - | - | - | - |
| \| RVA + other enteric bacteria \| \| --- \| | 6 (3.3) |  | 3 | 2 |  |  |  | 1 |
| + Salmonella | 2 (1.1) | - | 1 | 1 | - | - | - | - |
| + EAEC | 3 (1.6) | - | 2 | - | - | - | - | 1 |
| + ETEC | 1 (0.5) | - | - | 1 | - | - | - | - |

**Table S2** GenBank accession numbers assigned for all rotavirus genotypes based on gene segment VP4 and VP7 sequenced in this study.

| VP7 genotypes (G9) | | | |
| --- | --- | --- | --- |
| Accession Number | Strain | Accession Number | Strain |
| MT710743 | RVA/Human-wt/CHN/SX/2015/011/G9P[8] | MT710788 | RVA/Human-wt/CHN/SX/2017/457/G9P[8] |
| MT710744 | RVA/Human-wt/CHN/SX/2015/059/G9P[8] | MT710789 | RVA/Human-wt/CHN/SX/2017/459/G9P[8] |
| MT710745 | RVA/Human-wt/CHN/SX/2015/061/G9P[8] | MT710792 | RVA/Human-wt/CHN/SX/2018/004/G9P[8] |
| MT710746 | RVA/Human-wt/CHN/SX/2015/065/G9P[8] | MT710793 | RVA/Human-wt/CHN/SX/2018/010/G9P[8] |
| MT710747 | RVA/Human-wt/CHN/SX/2015/069/G9P[8] | MT710794 | RVA/Human-wt/CHN/SX/2018/026/G9P[8] |
| MT710748 | RVA/Human-wt/CHN/SX/2015/081/G9P[8] | MT710795 | RVA/Human-wt/CHN/SX/2018/064/G9P[8] |
| MT710749 | RVA/Human-wt/CHN/SX/2015/087/G9P[8] | MT710796 | RVA/Human-wt/CHN/SX/2018/065/G9P[8] |
| MT710750 | RVA/Human-wt/CHN/SX/2015/117/G9P[8] | MT710797 | RVA/Human-wt/CHN/SX/2018/074/G9P[8] |
| MT710751 | RVA/Human-wt/CHN/SX/2015/121/G9P[8] | MT710798 | RVA/Human-wt/CHN/SX/2018/087/G9P[8] |
| MT710752 | RVA/Human-wt/CHN/SX/2015/132/G9P[8] | MT710800 | RVA/Human-wt/CHN/SX/2018/095/G9P[8] |
| MT710755 | RVA/Human-wt/CHN/SX/2015/143/G9P[8] | MT710801 | RVA/Human-wt/CHN/SX/2018/109/G9P[8] |
| MT710756 | RVA/Human-wt/CHN/SX/2015/151/G9P[8] | MT710802 | RVA/Human-wt/CHN/SX/2018/117/G9P[8] |
| MT710757 | RVA/Human-wt/CHN/SX/2015/156/G9P[8] | MT710805 | RVA/Human-wt/CHN/SX/2018/322/G9P[8] |
| MT710758 | RVA/Human-wt/CHN/SX/2016/002/G9P[8] | MT710806 | RVA/Human-wt/CHN/SX/2018/329/G9P[8] |
| MT710759 | RVA/Human-wt/CHN/SX/2016/037/G9P[8] | MT710807 | RVA/Human-wt/CHN/SX/2018/357/G9P[8] |
| MT710761 | RVA/Human-wt/CHN/SX/2016/067/G9P[8] | MT710808 | RVA/Human-wt/CHN/SX/2018/391/G9P[8] |
| MT710763 | RVA/Human-wt/CHN/SX/2016/079/G9P[8] | MT710810 | RVA/Human-wt/CHN/SX/2018/400/G9P[4] |
| MT710764 | RVA/Human-wt/CHN/SX/2016/112/G9P[8] | MT710811 | RVA/Human-wt/CHN/SX/2018/412/G9P[8] |
| MT710765 | RVA/Human-wt/CHN/SX/2016/339/G9P[8] | MT710812 | RVA/Human-wt/CHN/SX/2019/003/G9P[8] |
| MT710766 | RVA/Human-wt/CHN/SX/2016/384/G9P[8] | MT710813 | RVA/Human-wt/CHN/SX/2019/006/G9P[8] |
| MT710768 | RVA/Human-wt/CHN/SX/2017/004/G9P[8] | MT710814 | RVA/Human-wt/CHN/SX/2019/013/G9P[8] |
| MT710771 | RVA/Human-wt/CHN/SX/2017/046/G9P[8] | MT710815 | RVA/Human-wt/CHN/SX/2019/026/G9P[8] |
| MT710772 | RVA/Human-wt/CHN/SX/2017/047/G3P[8] | MT710816 | RVA/Human-wt/CHN/SX/2019/044/G9P[8] |
| MT710773 | RVA/Human-wt/CHN/SX/2017/050/G9P[8] | MT710817 | RVA/Human-wt/CHN/SX/2019/045/G9P[8] |
| MT710774 | RVA/Human-wt/CHN/SX/2017/053/G9P[8] | MT710818 | RVA/Human-wt/CHN/SX/2019/063/G9P[8] |
| MT710775 | RVA/Human-wt/CHN/SX/2017/071/G9P[8] | MT710819 | RVA/Human-wt/CHN/SX/2019/101/G9P[8] |
| MT710778 | RVA/Human-wt/CHN/SX/2017/114/G9P[8] | MT710820 | RVA/Human-wt/CHN/SX/2019/102/G9P[8] |
| MT710779 | RVA/Human-wt/CHN/SX/2017/138/G9P[8] | MT710821 | RVA/Human-wt/CHN/SX/2019/116/G9P[8] |
| MT710780 | RVA/Human-wt/CHN/SX/2017/152/G9P[8] | MT710822 | RVA/Human-wt/CHN/SX/2019/118/G9P[8] |
| MT710784 | RVA/Human-wt/CHN/SX/2017/165/G9P[8] | MT710824 | RVA/Human-wt/CHN/SX/2019/339/G9P[8] |
| MT710787 | RVA/Human-wt/CHN/SX/2017/456/G9P[8] |  |  |

| VP7 genotypes (G1, G2 and G3) | | | |
| --- | --- | --- | --- |
| Accession Number | Strain | Accession Number | Strain |
| MT710762 | RVA/Human-wt/CHN/SX/2016/075/G1P[8] | MT710772 | RVA/Human-wt/CHN/SX/2017/047/G3P[8] |
| MT710753 | RVA/Human-wt/CHN/SX/2015/136/G2P[4] | MT710776 | RVA/Human-wt/CHN/SX/2017/080/G3P[8] |
| MT710754 | RVA/Human-wt/CHN/SX/2015/140/G2P[4] | MT710777 | RVA/Human-wt/CHN/SX/2017/082/G3P[8] |
| MT710760 | RVA/Human-wt/CHN/SX/2016/041/G2P[4] | MT710786 | RVA/Human-wt/CHN/SX/2017/271/G3P[8] |
| MT710785 | RVA/Human-wt/CHN/SX/2017/267/G2P[4] | MT710790 | RVA/Human-wt/CHN/SX/2017/472/G3P[8] |
| MT710809 | RVA/Human-wt/CHN/SX/2018/393/G2P[4] | MT710791 | RVA/Human-wt/CHN/SX/2017/473/G3P[8] |
| MT710823 | RVA/Human-wt/CHN/SX/2019/270/G2P[4] | MT710799 | RVA/Human-wt/CHN/SX/2018/089/G3P[8] |
| MT710767 | RVA/Human-wt/CHN/SX/2017/001/G3P[8] | MT710803 | RVA/Human-wt/CHN/SX/2018/144/G3P[8] |
| MT710769 | RVA/Human-wt/CHN/SX/2017/020/G3P[8] | MT710804 | RVA/Human-wt/CHN/SX/2018/146/G3P[8] |
| MT710770 | RVA/Human-wt/CHN/SX/2017/021/G3P[8] |  |  |

| VP4 genotypes (P[8]) | | | |
| --- | --- | --- | --- |
| Accession Number | Strain | Accession Number | Strain |
| MT710825 | RVA/Human-wt/CHN/SX/2015/011/G9P[8] | MT710875 | RVA/Human-wt/CHN/SX/2017/199/G9P[8] |
| MT710826 | RVA/Human-wt/CHN/SX/2015/013/G9P[8] | MT710876 | RVA/Human-wt/CHN/SX/2017/200/G9P[8] |
| MT710827 | RVA/Human-wt/CHN/SX/2015/059/G9P[8] | MT710878 | RVA/Human-wt/CHN/SX/2017/271/G3P[8] |
| MT710828 | RVA/Human-wt/CHN/SX/2015/060/P[8] | MT710879 | RVA/Human-wt/CHN/SX/2017/272/G9P[8] |
| MT710829 | RVA/Human-wt/CHN/SX/2015/061/G9P[8] | MT710880 | RVA/Human-wt/CHN/SX/2017/394/G9P[8] |
| MT710830 | RVA/Human-wt/CHN/SX/2015/063/G9P[8] | MT710881 | RVA/Human-wt/CHN/SX/2017/427/G9P[8] |
| MT710831 | RVA/Human-wt/CHN/SX/2015/069/G9P[8] | MT710882 | RVA/Human-wt/CHN/SX/2017/456/G9P[8] |
| MT710832 | RVA/Human-wt/CHN/SX/2015/081/G9P[8] | MT710883 | RVA/Human-wt/CHN/SX/2017/462/G9P[8] |
| MT710833 | RVA/Human-wt/CHN/SX/2015/087/G9P[8] | MT710884 | RVA/Human-wt/CHN/SX/2018/004/G9P[8] |
| MT710834 | RVA/Human-wt/CHN/SX/2015/117/G9P[8] | MT710885 | RVA/Human-wt/CHN/SX/2018/005/G9P[8] |
| MT710835 | RVA/Human-wt/CHN/SX/2015/121/G9P[8] | MT710886 | RVA/Human-wt/CHN/SX/2018/026/G9P[8] |
| MT710836 | RVA/Human-wt/CHN/SX/2015/132/G9P[8] | MT710887 | RVA/Human-wt/CHN/SX/2018/032/P[8] |
| MT710839 | RVA/Human-wt/CHN/SX/2015/143/G9P[8] | MT710888 | RVA/Human-wt/CHN/SX/2018/064/G9P[8] |
| MT710840 | RVA/Human-wt/CHN/SX/2015/144/G9P[8] | MT710889 | RVA/Human-wt/CHN/SX/2018/067/P[8] |
| MT710841 | RVA/Human-wt/CHN/SX/2015/149/G9P[8] | MT710890 | RVA/Human-wt/CHN/SX/2018/087/G9P[8] |
| MT710842 | RVA/Human-wt/CHN/SX/2015/151/G9P[8] | MT710891 | RVA/Human-wt/CHN/SX/2018/092/G9P[8] |
| MT710843 | RVA/Human-wt/CHN/SX/2015/156/G9P[8] | MT710892 | RVA/Human-wt/CHN/SX/2018/120/G9P[8] |
| MT710844 | RVA/Human-wt/CHN/SX/2015/157/P[8] | MT710893 | RVA/Human-wt/CHN/SX/2018/122/G9P[8] |
| MT710845 | RVA/Human-wt/CHN/SX/2016/001/P[8] | MT710894 | RVA/Human-wt/CHN/SX/2018/146/G3P[8] |
| MT710846 | RVA/Human-wt/CHN/SX/2016/002/G9P[8] | MT710895 | RVA/Human-wt/CHN/SX/2018/151/G9P[8] |
| MT710847 | RVA/Human-wt/CHN/SX/2016/004/P[8] | MT710896 | RVA/Human-wt/CHN/SX/2018/159/G9P[8] |
| MT710848 | RVA/Human-wt/CHN/SX/2016/022/P[8] | MT710897 | RVA/Human-wt/CHN/SX/2018/173/G9P[8] |
| MT710849 | RVA/Human-wt/CHN/SX/2016/039/P[8] | MT710898 | RVA/Human-wt/CHN/SX/2018/176/G9P[8] |
| MT710851 | RVA/Human-wt/CHN/SX/2016/056/G9P[8] | MT710899 | RVA/Human-wt/CHN/SX/2018/322/G9P[8] |
| MT710852 | RVA/Human-wt/CHN/SX/2016/058/G9P[8] | MT710900 | RVA/Human-wt/CHN/SX/2018/391/G9P[8] |
| MT710853 | RVA/Human-wt/CHN/SX/2016/067/G9P[8] | MT710902 | RVA/Human-wt/CHN/SX/2018/396/G9P[8] |
| MT710854 | RVA/Human-wt/CHN/SX/2016/073/G9P[8] | MT710904 | RVA/Human-wt/CHN/SX/2018/403/G9P[8] |
| MT710855 | RVA/Human-wt/CHN/SX/2016/075/G1P[8] | MT710905 | RVA/Human-wt/CHN/SX/2018/411/G9P[8] |
| MT710856 | RVA/Human-wt/CHN/SX/2016/112/G9P[8] | MT710906 | RVA/Human-wt/CHN/SX/2019/003/G9P[8] |
| MT710857 | RVA/Human-wt/CHN/SX/2016/124/G9P[8] | MT710907 | RVA/Human-wt/CHN/SX/2019/013/G9P[8] |
| MT710858 | RVA/Human-wt/CHN/SX/2016/172/G9P[8] | MT710908 | RVA/Human-wt/CHN/SX/2019/014/G9P[8] |
| MT710859 | RVA/Human-wt/CHN/SX/2016/339/G9P[8] | MT710909 | RVA/Human-wt/CHN/SX/2019/026/G9P[8] |
| MT710860 | RVA/Human-wt/CHN/SX/2016/384/G9P[8] | MT710910 | RVA/Human-wt/CHN/SX/2019/028/G9P[8] |
| MT710861 | RVA/Human-wt/CHN/SX/2017/001/G3P[8] | MT710911 | RVA/Human-wt/CHN/SX/2019/031/P[8] |
| MT710862 | RVA/Human-wt/CHN/SX/2017/002/P[8] | MT710912 | RVA/Human-wt/CHN/SX/2019/045/G9P[8] |
| MT710863 | RVA/Human-wt/CHN/SX/2017/004/G9P[8] | MT710913 | RVA/Human-wt/CHN/SX/2019/062/G9P[8] |
| MT710864 | RVA/Human-wt/CHN/SX/2017/046/G9P[8] | MT710914 | RVA/Human-wt/CHN/SX/2019/063/G9P[8] |
| MT710865 | RVA/Human-wt/CHN/SX/2017/047/G3P[8] | MT710915 | RVA/Human-wt/CHN/SX/2019/102/G9P[8] |
| MT710866 | RVA/Human-wt/CHN/SX/2017/048/P[8] | MT710916 | RVA/Human-wt/CHN/SX/2019/116/G9P[8] |
| MT710867 | RVA/Human-wt/CHN/SX/2017/050/G9P[8] | MT710918 | RVA/Human-wt/CHN/SX/2019/136/G9P[8] |
| MT710868 | RVA/Human-wt/CHN/SX/2017/052/P[8] | MT710922 | RVA/Human-wt/CHN/SX/2019/325/G9P[8] |
| MT710869 | RVA/Human-wt/CHN/SX/2017/079/P[8] | MT710923 | RVA/Human-wt/CHN/SX/2019/335/P[8] |
| MT710870 | RVA/Human-wt/CHN/SX/2017/083/P[8] | MT710924 | RVA/Human-wt/CHN/SX/2019/339/G9P[8] |
| MT710871 | RVA/Human-wt/CHN/SX/2017/114/G9P[8] | MT710925 | RVA/Human-wt/CHN/SX/2019/341/P[8] |
| MT710872 | RVA/Human-wt/CHN/SX/2017/157/G9P[8] | MT710926 | RVA/Human-wt/CHN/SX/2019/361/G9P[8] |
| MT710873 | RVA/Human-wt/CHN/SX/2017/163/G9P[8] | MT710927 | RVA/Human-wt/CHN/SX/2019/362/G9P[8] |
| MT710874 | RVA/Human-wt/CHN/SX/2017/165/G9P[8] |  |  |

| VP4 genotypes (P[4]) | | | |
| --- | --- | --- | --- |
| Accession Number | Strain | Accession Number | Strain |
| MT710837 | RVA/Human-wt/CHN/SX/2015/136/G2P[4] | MT710903 | RVA/Human-wt/CHN/SX/2018/400/G9P[4] |
| MT710838 | RVA/Human-wt/CHN/SX/2015/140/G2P[4] | MT710917 | RVA/Human-wt/CHN/SX/2019/118/G9P[4] |
| MT710850 | RVA/Human-wt/CHN/SX/2016/041/G2P[4] | MT710919 | RVA/Human-wt/CHN/SX/2019/270/G2P[4] |
| MT710877 | RVA/Human-wt/CHN/SX/2017/267/G2P[4] | MT710920 | RVA/Human-wt/CHN/SX/2019/271/P[4] |
| MT710901 | RVA/Human-wt/CHN/SX/2018/393/G2P[4] | MT710921 | RVA/Human-wt/CHN/SX/2019/284/P[4] |
